# Producing social honor through the hierarchy of study abroad: evidence from Chinese middle class

**DOI:** 10.3389/fsoc.2026.1780881

**Published:** 2026-06-12

**Authors:** Shasha Du, Michael Ohsfeldt

**Affiliations:** 1 School of Social and Public Administration, Lingnan Normal University, Zhanjiang, Guangdong, China; 2 Department of Social and Cultural Studies, Jiangsu Administration Institute, Nanjing, China; 3 Department of Psychological Sciences, Tarleton State University, Stephenville, TX, United States

**Keywords:** honor producing, hybrid social character, middle class, social stratification, study abroad hierarchy

## Abstract

Employing David Riesman’s theory of social character, this study examines how China’s middle class produces social honor through a hierarchically structured preference for overseas study destinations. Based on semi-structured interviews, our research identifies three distinct logics underlying honor production. The tradition-directed logic emphasizes pragmatism and collectivist values, the inner-directed logic centers on intergenerational cultural transmission, and the other-directed logic prioritizes consumption experience and social media visibility. These logics do not operate in isolation but interact within a hybrid framework, shaped by China’s compressed modernity. Key actors engage in strategic negotiations, collectively constructing honor as a dynamic and contested consensus. This study moves beyond frameworks of conspicuous consumption and cultural capital by revealing the socio-psychological mechanisms through which honor is actively manufactured, offering new insights into how middle-class actors navigate globalization, identity, and stratification.

## 1 Introduction

Amid global economic and cultural integration, Chinese middle-class students increasingly pursue overseas studies for academic advancement and cultural exposure. Empirical trends underscore this phenomenon. Cumulative outbound students reached 6.56 million from 1978 to 2019 (Ministry of Education of the People’s Republic of China, 2020), with annual figures rising to 803,000 by 2024—a 2.1% year-on-year increase (Center for China and Globalization, 2025). Over 55% originate from middle-class households, and notably, public-sector employees constitute more than 62% of their parents (Kantar Group, 2022, p. 7), establishing this demographic as the dominant force in international education. Destination preferences reveal stratified patterns: the United States (28.92%) and United Kingdom (14.27%) remain predominant choices, followed by Australia (9.15%) and Canada (7.81%), while South Korea, Japan, and Southeast Asian nations collectively attract around 10% of students (Center for China and Globalization, 2024).

This stratification is mirrored in China’s social discourse. The widely circulated post “The Discrimination Chain of Chinese Students Studying Abroad” (Nao, 2021), in Zhihu which is China’s largest Q&A-style online community, exceeded 812,000 views after dissemination across major platforms including WeChat and Tiktok. This discussion classifies destinations into three distinct categories: premier destinations including the United States and United Kingdom; secondary destinations comprising Canada, Australia, Western Europe, Japan, and South Korea; and peripheral destinations encompassing Southeast Asia, Russia, and Eastern Europe. This discourse permeates middle-class social domains—employment, marriage markets, networking—suggesting that destination choice transcends educational or economic calculation to embody implicit valuations of social honor.

Social honor, a publicly recognized status conferring prestige within a specific social field (Weber, 1978), is often analyzed through the lenses of Veblen’s (1899) conspicuous consumption or Bourdieu’s (1986) cultural capital. At its core, it constitutes a sophisticated construction of aesthetic preferences based on hierarchized distinctions (Wohl, 2015). Social honor is thus shaped by specific social structures, collective habits of interaction, and value-laden aesthetics. While these frameworks explain the display and institutionalization of distinction, they offer limited insight into the active processand socio-psychological mechanisms through which social groups collectively produce, contest, and validate honor. How is the symbolic value of an overseas degree manufactured and made desirable? What are the underlying logics that transform an educational pathway into a vehicle for honor?

This study addresses this gap by investigating the production process of social honor. We argue that for China’ s middle class, honor construction operates not exclusively through economic display or passive capital accumulation, but as a strategic, agentic process rooted in a class-specific socio-psychological habitus. This habitus navigates competing logics of value within a context of compressed modernity (Kyung-Sup, 2022). To address this gap, the underexplored process and socio-psychological mechanisms of honor production, our study employs David Riesman et al.’s theory (1950, p. 24) of social characters. By distinguishing between various social characters, the theory allows us to decode the different logics through which actors interpret, pursue, and legitimize social honor. It posits that what is considered honorable, and how one performs to achieve it, depends on whether one seeks validation from established traditions, internalized principles, or contemporaneous peer groups.

Accordingly, this study examines three research questions: First, what hierarchy of overseas education preferences exists among China’s middle class? Second, what systems of social honor underlie these preferences? Third, through what mechanisms and by which actors is such honor actively produced? Employing Riesman’s Theory of Social Character, we analyze the production mechanisms of social honor embedded within educational preferences. We posit that middle-class honor construction operates neither exclusively through conspicuous consumption, nor cultural capital accumulation, but rather functions as a class psychosocial habitus establishing distinctions within specific social characters.

## 2 Literature review and theoretical background

### 2.1 Study abroad as conspicuous consumption

Overseas education constitutes a significant field for status competition. It can be understood as a form of conspicuous consumption, where families convert economic capital into visible signs of distinction (Veblen, 1899, p. 22; Veblen, 1915a,b, 2015). Contemporary research shows this educational consumption is less about sheer waste and more about signaling alignment with global, elite norms—a performance of “global parenting competence” or cultural fluency (Zhang and Tao, 2015). The substantial cost barrier also creates exclusivity, transforming international study into rare social status (Chiramba and Motala, 2023). This status is performative; returnees strategically exhibit cultural artifacts or linguistic competencies to gain access to elite circles (Brooks and Waters, 2023). Thus, conspicuous consumption generates honor by signaling alignment with elite practices to maintain social recognition and solidify class position (Mestrovic, 1997, 2003).

Empirical evidence demonstrates this mechanism: Zhang and Tao (2015) found Shanghai families spending $84,000 annually on international schools primarily (67%) sought to display “global parenting competence” within social networks, with only 23% prioritizing educational returns. Such consumption exhibits theatrical stratification effects. While working-class families pursue vocational training at community colleges, elites engage in cultural distinction through liberal arts education. Lamont (2022) documented how Ivy League parents view classical literature degrees as “aristocratic lineage certification”, constructing cultural honor through symbolic expenditures like Latin language courses.

A substantial body of public data also lends indirect support to existence of overseas education as a form of conspicuous consumption. The higher the cost of education in a study destination, the more it attracts applicants rather than deterring them. Nearly 25% of the 6,628 participants have chosen the United States as the destination for studying abroad which is the only country with more than 20%. Countries with a proportion between 10 and 19% are the United Kingdom, Canada, and Australia. Countries with a proportion between 2 and 9% include Japan, France, Germany, Singapore, and New Zealand. Other countries, including Russia, Malaysia, Thailand, South Korea, etc. are less than 1% (Kantar Group, 2022, p. 58). For the 10th consecutive year, China has become the largest source of international students in the United States^1^ and this number has increased by more than 30 times between 1979 to 2018, from 1,000 to 369,548. Even in the era of covid-19, China has become the largest leading place origin in 2020 both at intensive English programs and international scholars in the United States.^2^

### 2.2 Educational credentials as cultural capital

Overseas education constitutes a strategic field for status competition and capital conversion. From a Bourdieusian perspective, it is a key site where economic capital is invested to acquire institutionalized cultural capital—the academic credential—which in turn can be reconverted into enhanced social and economic advantages (Bourdieu, 1986). This process is deeply embedded in primary socialization, where familial habitus orient future investments (Bourdieu and Passeron, 1990). During this period, parents’ judgments regarding educational honor begin to shape their children’s inner values. Specifically, academic qualifications constitute an institutionalized form of cultural capital (Bourdieu, 1986), embodying Weber’s (1978) concept of “social honor”—an identity marker validated through authoritative certification. This symbolic capital emerges from institutional monopolies: Harvard Law School ritualizes discursive training into “critical thinking ceremonies”, enabling graduates to monopolize 86% of partnerships in elite U.S. law firms through exclusive narrative frameworks (Jewel, 2008). Prestigious institutional labels similarly function as state-endorsed credentials, with graduates enjoying 2.3 times higher public-sector recruitment rates, reflecting administrative power’ s direct validation of academic honor (Tian and Liu, 2024).

When transformed into cultural capital, academically generated honor permeates social life. Gaddis (2015) longitudinal research revealed Ivy League diplomas significantly exceed their economic value in marriage markets—holders show a 3.7-fold increase in elite marital alliances, forming exclusive “honor exchange circuits” based on cultural distinction. Institutional collusion in status closure manifests when the UK’ s Institute of Chartered Accountants excludes non-Russell Group graduates from accelerated promotion, despite equivalent professional examination scores (Friedman and Laurison, 2019), exposing systemic alignment between academic honor and social closure mechanisms.

### 2.3 The honor-orientation in traditional Chinese educational philosophy

Within the framework of Confucian educational philosophy, higher learning has long been regarded as a source of honor. This conception of honor permeated all social strata, a notion succinctly captured by the ancient adage: “All occupations are base; only the pursuit of learning is exalted”.^3^ More specifically, traditional Chinese higher education emphasized two principal orientations. The first was a pragmatic orientation towards learning. The higher education theory of *Jingshi Zhiyong* (经世致用) in China is the best representative of this kind of ethical spirit in Confucianism rising in the 1600s. It means that elite class always hold a world outlook that knowledge and theories should be paid attention to social realities, face contradictions, and use what they have learned to solve social problems, in order to achieve the social solidarity and public security, with against the unrealistic emptiness (Schulte, 2012; Yang, 2016). This trend of thought was promoted by Confucians such as Zongxi Huang and Fuzhi Wang. *Jingshi Zhiyong* is slightly different from Veblen’s theory on leisure, honorific, and industry in Western ancient society. The usefulness of knowledge learning has become a commom goal of traditional higher education in ancient China.

Collectivism is another core of ancient Chinese Confucian ethics for higher education. It advocated that the purpose of education for the Chinese was to govern the state and rejuvenate the family. From the claim of “Ren” (仁) (Møllgaard, 2015; Ramsey, 2016), which means full of benevolence to others, by Confucius, “*Xiushen Zhiguo*” (修身治国) (Qiu, 2010), which means self-cultivation and devoted to nation, proposed by Zhongshu Dong, to the thought of “*Jingshi Zhiyong*” (经世致用), which means the rules of governing by nobility should be based on social realities and public interests, and rather than the Utopian conceive by personal preference. These classic theories are all in emphasizing collective interests and honor in a kind of cross-class unity, not just limited to the leisure class. During all this time, the country always emphasizes that we are here to be useful to the society rather than to be individual free and enjoyment (Earley, 1989; Winfield et al., 2000; Cao, 2009).

### 2.4 Social honor and middle class

Social honor as a collectively validated, desirable status—a positive social estimation of prestige—that is actively sought, conferred, and contested within a specific social field (Weber, 1978). It is the symbolic value that makes one destination or degree more esteemed than another. This requires distinguishing honor from social honor. Honor can be a personal or familial attribute, whereas social honor is inherently inter-subjective; it requires public recognition and validation by a relevant group or community (Schulte, 2012). It is not merely possessed but is continually performed and affirmed through social interaction. Social honor is embodied and signaled through various carriers. It can be objectified in cultural artifacts, institutionalized in professional titles or elite employment, and embodied in dispositions, accents, or manners (Reed and Johnson, 2023). Its value lies in its exclusivity and the shared understanding of what it represents.

The middle class, situated between the working class and the elite, is characterized by a distinct relationship to honor and status. Bourdieu’ s analysis of the French petite bourgeoisie reveals a class fraught with status anxiety—a reverence for and anxious imitation of highbrow culture, which they perceive as a path to legitimacy and social elevation (Bourdieu and Passeron, 1990: 39–42). For the middle class, honor is not an ascribed birthright nor solely a function of economic power; it is a precarious achievement, often sought through the acquisition and display of institutionalized cultural capital, particularly educational credentials (Ponzini, 2020).

This creates a relentless drive for distinction—the symbolic demarcation of boundaries from those below and aspirational alignment with those above. Lifestyle choices, consumption patterns, and, most critically, educational strategies become primary arenas for this status competition (Bennett et al., 2009). The middle-class habitus is thus oriented towards accumulation, investment, and the strategic conversion of capital to secure and enhance social honor (Singh, 2024).

### 2.5 Research gap

Existing studies identify two sociological explanations for middle-class study-abroad behaviors: First, as a form of conspicuous consumption, overseas education enables status demonstrations through emulation of higher classes and claims of exclusivity, thereby producing social honor. Second, as cultural capital, international degrees function as institutionalized markers of distinction through authoritative certification. This capital subsequently permeates various aspects of middle-class life.

Nevertheless, both explanatory frameworks exhibit critical blind spots: First, they fail to unveil the socio-psychological dispositions through which social classes generate honor, leaving the meaning-making frameworks of class groups underexplored. Second, they inadequately address why these honor systems emerge, neglecting analyzes of the operational mechanisms underpinning such hierarchies.

This study thus employs social character theory to investigate the socio-psychological foundations of honor production within China’s middle class, decoding the mechanisms within social structures. Simultaneously, it identifies the principal actors constructing such systems to clarify the typology of social character shaping honor production.

### 2.6 Theoretical framework

Riesman et al.’s (1950, p. 7) theory of social character also sees the characteristics, drives, and behaviors change within societies over time as their dynamics development. Each ideal type of social character outlined by Riesman et al. (1950, p. 9) focuses on a form of direction, with tradition-directed, inner-directed, and other-directed people and societies. In traditional direction “the conformity of the individual tends to reflect his membership in a particular age-grade, clan, or caste.” (Riesman et al., 1950, p. 10). They are intended to replace and replicate those who came before and are staunchly bound to their guiding traditions. Then inner-directed have a strong socialization process in the sense that it is implanted early in life by the elders and directed toward generalized but nonetheless inescapably destined goals (Riesman et al., 1950, p. 13), so though they have a distinct perspective and values placed within them, this is a resilient direction intended to keep directing them no matter where they are or what they face. Those who are other-directed are constantly beholden to the jury of their peers, tirelessly seeking their ever-changing approval (Riesman et al., 1950, p. 19). The central metaphor for the other-directed is that of the radar due to their guidance being in response to many external standards, while the inner-directed is that of the gyroscope for their guidance coming from a singular internal standard.

At its core, social character theory aims to uncover how dominant value orientations and behavioral norms are formed in specific societal periods. Each type of social character corresponds to a distinct conformity. Tradition-directed societies adhere to inherited customs and hierarchical order, inner-directed societies follow internalized ideals and personal goals, while other-directed societies are attuned to the expectations and feedback of peer groups. These principles are not merely abstract. They constitute the foundational framework of a society’s mechanisms of honor production. This framework determines which behaviors and achievements are accorded honor, by whom they are recognized, and how they are symbolically converted. Within this structure, social resources associated with honor are allocated. Those who conform to honor-based norms gain greater access to collective resources—such as money, status, and knowledge (Pratto et al., 1999). Therefore, understanding the dominant social character and its corresponding principles of compliance provides the theoretical starting point for systematically analyzing the logic of honor production in a given society.

These three types of social character correspond to three distinct societal eras: pre-industrial, industrial, and consumer society. Through nearly four decades of rapid modernization, China has intricately blended these three forms. As a result, all three social characters now coexist in contemporary Chinese society (Zhou, 2024). According to Zhou’s perspective, in Western societies, these three character types emerge diachronically in sequence. However, due to China’s later-start yet much more rapid modernization process, a form of “condensed modernization” has taken shape. This has led to the three social character types not replacing one another successively, but rather coexisting simultaneously in contemporary Chinese society. On the one hand, the values and behavioral patterns of traditional society have not yet completely faded. On the other hand, the individualism and rational spirit of industrial society, along with the consumerism and other-directed orientation of post-industrial society, have rapidly flowed in. Owing to China’s culture of filial piety, populations representing these three social character types are closely bound within a shared family space. This coexistence leads the middle class, for instance, to combine familial support with personal choice in major decisions such as home purchasing and education. The concurrent presence of these multiple character types results in highly blended and strategic behavioral patterns within the middle class. This coexistence constitutes a social fact that must be acknowledged in any analysis of China’ s mechanisms of honor production.

In regard to Veblen, it is important to note that Riesman et al. (1950) saw inner-direction in the conspicuous consumption of Veblen’s era as the Veblenese conspicuous consumer is seeking to fit into a role demanded of him by his station, or hoped-for station; whereas the other-directed consumer seeks experiences rather than things and yearns to be guided by others rather than to dazzled them with display.” (Veblen, 1899, p. 118) This is an important point as what constitutes conspicuousness and its displays changes over time with social character type. When the theories are combined and compared, the tradition-directed conspicuous displays were what Veblen (1899, p. 120) refers to as booty, the spoils of conquest and plunder. For the inner-directed conspicuous displays involve wealth and the ability to waste it, whether through leisure or later consumption as Veblen details. Yet for the other-directed and the heightened need for approval from others, conspicuous displays entail experiences, that you did what was in fashion or what others have not yet or could not. Riesman (1953) also wrote a book on Veblen, reinforcing the importance of such theories within his own.

Unlike Veblen’s framework, honor in Bourdieu’s sense operates as symbolic capital. It is exchangeable with economic, cultural, and social capital. This understanding rests on the underlying premise that symbolic capital is inherently practically useful. Its significance thus extends beyond mere class distinction; it also functions as an instrument through which social actors access and exchange resources within society. Education, as a form of cultural capital, offers a clear example of such exchange with honor. When overseas higher education functions as a form of honor, it serves at least two key purposes. First, it acts as a marker of class status. Second, it operates as a tool for converting into other forms of capital.

**Figure 1:**
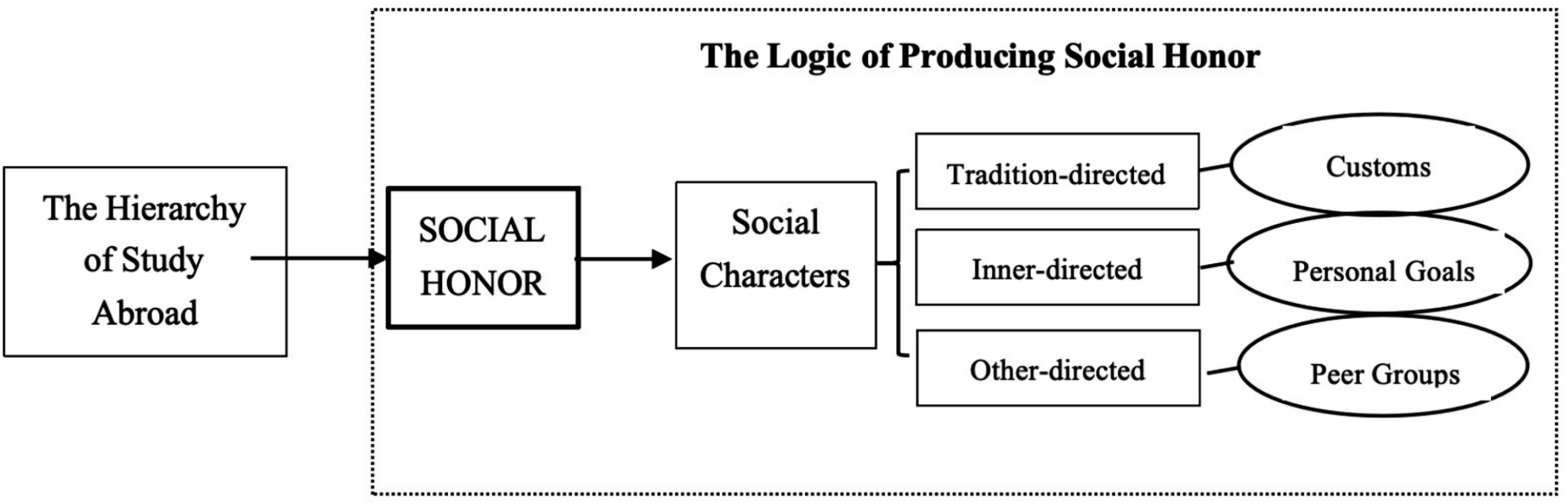
Theoretical framework of social honor producing via the hierarchy of study abroad.

This paper defines honor as a form of social recognition rooted in the shared norms and values of a particular class or group. It confers prestige, dignity, and legitimacy upon individuals or collectives, and can be converted into tangible social resources and agential capacity. The question we should explore is: under what circumstances is it produced as a form of conspicuous display, and under what conditions is it produced as a practical instrument? Alternatively, in certain contexts, both functions may be blended and utilized by social actors. Therefore, clarifying the production mechanism of social honor is evidently more crucial. From the perspective of different socio-psychological logics, it is fundamentally shaped by the dominant social character. Drawing on social character theory, this paper will examine the production mechanisms of honor in the context of middle-class overseas study across three socio-psychological logics, aiming to illustrate how conspicuous display and practical utility are intertwined in social practice (see Figure 1). The choice of study abroad destinations is, in essence, a process of producing honor. This honor, guided by the action rules associated with different social character types, is recognized either as conspicuous display or practical capital. These action rules serve as the key variables in analyzing the production of honor. They include tradition, personal goals, and peer groups.

## 3 Methodology

### 3.1 Research design and justification

This study employs a qualitative research design based on in-depth, semi-structured interviews. The research questions demand an exploration of the processes, meanings, and mechanisms behind honor production. Qualitative methods are uniquely suited to uncover the subjective interpretations, strategic reasoning, and lived experiences through which actors construct social reality, aspects that quantitative surveys of outcomes cannot fully capture (Glaser and Strauss, 1967).

### 3.2 Sampling and recruitment

The recruitment of participants was conducted over a period of 3 months (April–June 2023) and followed a multi-stage, iterative process designed to ensure the sample met the study’s strategic criteria for maximum variation and theoretical richness. Potential participants were initially identified and contacted through a combination of channels to access diverse networks within the Chinese international student population. These channels included university network, social media & online communities, and snowball sampling. We finally conducted 21 semi-structured interviews with Chinese students from middle-class families who had at least 6 months of overseas study experience across 13 countries (see Table 1). The sample was designed for maximum variation in destinations to capture the full spectrum of the perceived hierarchy. Meanwhile, 21 interviewers is the result of reaching theoretical saturation - the point at which collecting additional data no longer yields new thematic insights or conceptual categories relevant to our research questions (Strauss and Corbin, 1990). Middle class was targeted as the group most actively engaged in and anxious about status project of educational migration (Beardsmore, 2022). A minimum of 6 months was set to ensure participants had substantive immersive experience to reflect upon. Parents’ occupations were recorded as a key proxy indicator to prove they are indeed middle class. Interviews were conducted from April to June 2023, with follow-ups in July 2024 and 2025, following the principle of theoretical saturation. Interviews ceased when new data no longer yielded novel insights or categories.

**Table 1:** Information of interviewees.

Name	Country	Major	Types of study	School	Months of studying abroad	Occupation of parents
ZYN	U.S.	Anthropology	Visiting scholar	Boston University	14	Civil servants
WJX	U.S.	Mathematics	Doctor	Johns Hopkins University	7	Lawyer/doctor
	Canada		Master	Queen’s University	25	College professors
SY	U.S.	Business	Undergraduate	George Washington University	30	Doctor/business manager
ZYY	Philippines	Business	Undergraduate	Far Eastern University	26	Civil servants
MJY	U.K.	Business	Master	UCL	18	Civil servants
CXZ	Indonesia	Anthropology	Visiting scholar	University of North Sumatra	12	Doctor/college professor
QJF	France	Sociology	Undergrduate	EHESS	10	Business managers
JC	Germany	Sociology	Visiting scholar	University of Göttingen	11	Lawyer/financial manager
FH	Australia	Social policy	Visiting scholar	University of New South Wales	12	Bank manager/college professor
ZYL	Japan	Communication	Doctor	Kyoto University	32	Civil servant/middle school teacher
XCQ	U.S.	Electronic engineering	Doctor	University of California, Santa Barbara	21	Civil servants
LGG	U.K.	Business	Undergraduate	Newcastle University	40	Business managers
CDX	France	Literature	Visiting scholar	Université Paris-VIII	11	Business managers
HS	Italy	Arts and design	Undergraduate	Sapienza University of Rome	29	Business managers
XWS	U.S.	Sociology	Master	University of California, Berkeley	18	Middle school teacher
FXL	U.K.	Communication engineering	Doctor	University of Southampton	30	Middle school teacher
JL	U.K.	Civil engineering	Doctor	University of Bristol	17	Bank manager/college professor
XZ	U.S.	Mechanical engineering	Master	Texas A&M University	10	Doctors
JH	U.S.	Computer science	Doctor	Texas A&M University	10	Civil servants
	Singapore		Master	National University of Singapore	24	Civil servants
SHR	U.S.	Computer science	Master	Texas A&M University	21	Civil servants
GJP	U.K.	Statistics and business	Doctor	LSE	15	Civil servants

### 3.3 Data collection and analysis

The interview guide was structured around our research questions and theoretical framework: (1) Motivations for country choice (linking to hierarchy and logic). (2) Perceptions of differences between destinations (hierarchy). (3) Views of family, friends, and society on their overseas status (honor validation). (4) Life changes attributed to studying abroad (capital conversion). (5) Emotional experiences of the sojourn (aesthetic sensibility). The specific questions of this interview is as follows: (1) Could you please talk about why you choose the current country to study aboard? (2) Please talk about what are the differences among several worldwide popular study abroad destinations for Chinese students? (3) Please talk about your family, friends, spouse, classmates, or colleagues’ views on your overseas study status and country? (4) What changes do studying abroad experience provides for you in your current life or after returning to China? (5) What do you think about Chinese culture, society, and public opinion about your study abroad status and country? (6) As an overseas student in your current country, how do you feel? Interviews were transcribed verbatim and analyzed using a hybrid approach, combining deductive coding based on our theoretical framework with inductive thematic analysis to capture emergent narratives. Data presentation below integrates analytic commentary with illustrative extracts, with participants identified by pseudonym, gender, study level, and host country to provide context.

## 4 Findings

This paper will first introduce the hierarchy of study abroad destination choices among China’s middle class and examine in detail the national composition within each level. We will then explore, from the perspective of tradition-directed, inner-directed, and other-directed social characters, the production mechanism behind this hierarchy of study destinations as an honor system. Finally, various social actors participating in this creation of honor system will be thoroughly discussed.

### 4.1 The hierarchy of study overseas in China

The hierarchy of study abroad refers to a series of prior choices Chinese students to choose which countries to conduct their abroad higher education including undergraduate, graduate research or academic visiting activities. The hierarchy is a chain of preferences that to be based on comprehensive cultural evaluations of the destinations, which is full of affective reactions like pride, admiration, jealousness, prejudice, or even a sense of inferiority. According to the interviews we conducted, the hierarchy is divided into three levels based on honor as well as numbers of Chinese students and scholars. On the very top of it is the United States and UK. The second echelon consists of Canada, Western European countries like France and Germany. The third echelon includes Australia, Japan, Singapore, New Zealand, South Korea, Russia, Eastern European and other Asian countries, such as, Thailand, Indonesia, Malaysia, etc. The distinct hierarchy of study abroad destinations is evident in the following statements from participants XWS, LGG, and SHR:

“*The day I got the offer from a school in the U.S., our family WeChat group was flooded with messages saying I brought honor to my ancestors. My parents’ voices even went an octave higher with pride. One year later, at a new year gathering, when my high school friend mentioned studying in the Philippines, the dinner table fell silent, and the conversation quickly shifted to ‘Is it safe there for Chinese? The tuition is sure cheap, though’.*” (XWS-2407).^4^

*If you go to the United States, you will win praise and envy. If it is Australia, everyone feels a little bit confused, and say I do not know any colleges out there, except wild animals and farms.”* (LGG-2306).

“*Everyone will think you are crazy once you had an idea to a African college, it is better to go to any other universities in China rather than be killed or robbed at there*.” (SHR-2507).

Chinese students will choose their overseas universities or colleges mostly based on academic performances of each specific subjects and a comprehensive strength of schools. However, more interesting, cultural attraction of a country is more vital for them, if universities being chosen are at the same worldwide academic level relatively. The choices of these aboard locations are followed by the canon of honorific expenditure (Veblen, 1899) among various middle classes in China. The selection of international schools by middle-class families is driven not only by economic returns on education but, more significantly, by the pursuit of social honor. Such honor functions as a psychological habitus (Bourdieu, 1986) that serves to demarcate and reinforce class distinctions. A Chinese student studying in the U.S., GJP, noted that studying abroad is not merely a calculation of financial investment, but also the continuation of social connections and honor.

“*I did my undergrad in the U.S., and the whole four years probably cost around 2 million RMB. Back in China, I might only make about 100,000 a year. Do the math—that’s 20 years just to break even. But here’s the thing: it was never just a financial investment. It’s more about family pride and my own personal achievement. Besides, the children of my parents’ friends all went to the U.S. or the U.K. If I had gone to another country, it would have been hard for me to engage in their social conversations or tap into their alumni networks. After returning to China, I would have practically been out of that social circle.*” (GJP-2407).

In some extreme cases, Chinese students are more willing to choose universities in the United States, United Kingdom, and Canada than other countries even though the academic performances are not as good as them (Kumar et al., 2022). This effect will be amplified when we discuss which country they should study aboard publicly in some social occasions like festivals, weddings, and birthday parties. At this time, studying abroad has become a kind of conspicuous capital in collective behavior and value judgment among your neighbors, relatives and friends. Therefore, it becomes a kind of conspicuous activity participating in social interactions and relationships by actors, integrated into local interpersonal structures and ethics in China. When discussing how the choice of study destination often relates to social displaying, one international student remarked:

“*At Festival gatherings, elders and neighbors always like to ask where we studied abroad. Those who went to developed countries always receive more social attention. This attention can potentially lead to new social network connections, especially if everyone went to the same developed countries. But if you studied in Southeast Asia or Africa, you practically become the conversation stopper of the gathering. You might even be ignored in the following conversations.*” (XCQ-2306).

### 4.2 The logic of honor producing in middle class

Social honor, as constructed through the hierarchy of overseas study destinations, is often viewed as a form of conspicuous consumption. Alternatively, it can also function as cultural capital, convertible under certain social conditions into other forms of capital through its symbolic value. The core issue, however, lies in the need to further examine the active production mechanism of honor—the social process that endows it with recognizability, desirability, and efficacy. Unlike other social classes, the construction of honor within the middle class is particularly complex. To address this, this study employs the theory of social character, proposing three ideal-typical mechanisms for the generation of social honor. By analyzing the hierarchical patterns observed in the study destination choices of the middle class, the research identifies three distinct logics driving the production of honor. This helps explain why countries like the United States and the United Kingdom occupy the top tier of this hierarchy, while destinations in Southeast Asia and Africa remain at the lower end. It is important to note that these three ideal-typical mechanisms are highly abstract analytical constructs. In the actual practice of honor production, they are not strictly independent but consistently operate in a hybrid manner.

#### 4.2.1 Tradition-directed logic of honor producing: pragmatism and collectivism

For China’s middle class, pragmatic considerations serve as a crucial filter in selecting study-abroad destinations. This aligns with the traditional Chinese educational principle of *Jingshi Zhiyong*—applying knowledge for real-world impact. This utilitarian ethos has been effectively preserved and reinforced through China’s rapid modernization. The central question then becomes: how is this pragmatic approach to education constructed and validated as a form of social honor?

Honor is partially anchored in enduring communal norms, rituals, and inherited status (Krause et al., 2020, pp. 61–63). Following Riesman’s framework, these historically derived principles continuously shape the lifeworld of contemporary groups through behaviors and values. Its value stems from conformity to established hierarchies and visible fulfillment of traditional roles. Individual conduct is guided, clockwork-like, by fixed, transmitted traditions (Riesman et al., 1950, pp. 21–23). Life unfolds as if completing a predetermined circle on this clock face, emphasizing the fulfillment of a prescribed cycle. Under this tradition-directed logic, Chinese middle class tend to select study destinations that offer higher practical value after returning home. This value manifests concretely in employment prospects, social network building, and acquisition of knowledge, as well as skills. Thus, the Chinese middle class does not merely regard overseas education as a form of conspicuous consumption. XWS believes that the value of an overseas diploma lies primarily in its pragmatic utility, such as in job seeking, income, and social security benefits.

“*Going abroad must be learning technology, or management experience. What’s the purposes of learning literature and art? I do not know……but I did know, after returning to China, most students still face looking for jobs. Those who study art and literature are eager to be teachers in middle schools or universities as they will have stable wages. They are state-owned people, and the country will support you for a lifetime.*” (XWS-2407).

The United States as the top choice for China’s middle-class students is attributed to its pragmatic approach to education and superior quality of its academic institutions. The emphasis in American academia on efficiency, specialization, and codified standards aligns with the Confucian principle of *Jingshi Zhiyong*. Both traditions share a reality-based disposition, one that values actionable knowledge and systematic problem-solving. Thus, the Chinese middle class’ s attraction to American education is less a departure from local values than a recognition of shared pragmatic sensibilities.

Furthermore, the recognized high quality of American higher education grants its degrees considerable prestige within China. Graduates from U.S. institutions are often preferred by governments, enterprises, and universities alike. This preference stems from two interrelated perceptions. The academic excellence of American universities is widely acknowledged in China. Chinese middle class defines this excellence in terms of abundant research resources, high-impact publications, and prestigious accolades such as the Nobel Prize, Pulitzer Prize, and Turing Award. Second, American graduates are perceived to possess stronger professional competencies, a view attributed to the competitive admissions standards and rigorous academic training that characterize U.S. degree programs. Regarding the practical utility of academic performance and educational intensity in institutions of the host country, an international student remarked:

“*If you graduated form a oversea university ranked in the world top 200, according to Shanghai government, you can directly register for Shanghai residence. And about half of those top universities are probably in the United States. U.S. diploma simply carries more weight. Plus, those who come back from the U.S. often do stand out more in terms of practical skill.”* (SY-2306).

For second-tier institutions, the perceived utility of their degrees within China falls slightly below that of U.S. credentials. This utility, nonetheless, is still recognized across three dimensions: employment, social networking, and knowledge acquisition. For instance, British graduates are often viewed as having moderate employability, attributed by the middle class to the perceived lower intensity of student training. Similarly, universities in Canada, Australia, and Japan are seen as offering less competitive academic platforms due to their relatively modest research outputs. In contrast, degrees from third-tier countries are largely dismissed as lacking practical value. They are perceived to lag significantly behind their higher-tier counterparts across all noted criteria—institutional prestige, pedagogical rigor, and graduate employment. Three participants discussed how international students from different tiers of study destinations clearly differ in their practical value in the job market upon returning to China.

*“The UK actually has a lot of excellent universities, but its one-year master’s programs have really damaged their reputation. Nowadays, many big companies in China aren’t very keen on hiring these UK master’s graduates. It’s mainly because their professional skills are generally seen as weak. If you hire them, they often cannot solve real problems for the company. Think about it—what kind of talent can you really cultivate in just one year? The time is just too short.”* (MJY-2507).

*“Canada, Australia, and Japan are more like my second choice. If I cannot go to the U.S., I’d go to one of these countries. The thing is, universities there are seen as having average academic performance, and there aren’ t that many alumni back in China, so the quality of the professional networks you can tap into isn’ t that strong.”* (XZ-2407).

*“These days, even top universities in China aren’ t hiring PhDs from Southeast Asia anymore. The reason is simple: their teaching and publication abilities really aren’ t up to standard. Indonesian universities are actually very easy to enter. You do not need much talent and effort. I feel that both teachers and students are not doing their jobs properly. As I mentioned before, they are more likely to enjoy their life than academic performance.*” (CXZ-2507).

However, Chinese pragmatism is characterized by a collectivist orientation rather than individualism, representing its most distinct divergence from Western pragmatic traditions (Kolstad and Gjesvik, 2014). Its defining feature lies in the expectation that utility must extend its benefits to family, community, and the nation as a whole. More specifically, the most salient distinction between Chinese and Western societies in defining honor lies in its fundamentally collective nature (Yang et al., 2024). In Chinese context, the significance and purpose of an individual’ s achievements derive largely from their contribution to and recognition by collective entities—such as the clan, family, social network, organization, or nation (Lewis, 2021). Within this framework, honor is validated primarily when it engenders pride among one’s collective group, while personal sentiment remains secondary. This collectivist orientation helps explain the appeal of American-style individualism, liberalism, and personal hedonism in contemporary China, as they offer a contrasting cultural paradigm. Participant SY emphasized the importance of diligently contributing to the family as a collective after obtaining an overseas diploma.

*“Many advantages can be obtained for you and your family if you got a prestigious college certificate from overseas. You also can have the most excellent social insurances in China after your retirement. Your child can go to a better school, and there are advantages in finding a job as many companies need a Shanghai residence card to get in.*” (SY-2306).

#### 4.2.2 Inner-directed logic of honor producing: intergenerational transmission of cultural influence

The choice of study-abroad destinations among China’s middle class is closely tied to parental aspirations. When such aspirations are realized across generations, they are perceived as a form of social honor. This production of honor is intimately linked to what Riesman termed the inner-directed character. In this type, authority is already internalized within the individual. During childhood, parents act as “programmers,” instilling a set of clear, stable goals, values, and ideals—such as “achieving accomplishments,” “pursuing truth,” or “self-disciplined striving”—into the child (Riesman et al., 1950, p. 113). Once this internal psychological gyroscope is established, the individual primarily acts in accordance with this inner voice. At this stage, reverence for parents is transformed into loyalty to the internalized values derived from them. Social honor is subtly reproduced through the early familial implantation of a specific “honor symbol system” into the child’s personality structure, enabling them to continuously and autonomously enact a logic of honor production throughout their life. A student spoke about the crucial role their parents played in their decision to study abroad.

“*Following in my parents’ footsteps is kind of a common ethical rule for how kids should grow up. Our parents raised us, spending both money and time—so we should really think their wishes over. These two years in the U.S. have cost my parents a pretty penny, but they’re happy. They’re happy, and that makes me happy too. Because I’ve made their dream come true. And of course, it’s my dream as well.*” (ZYN-2306).

Middle-class offspring’s study-abroad destination choices are heavily influenced by parental preferences. These preferences transmit intergenerationally through parenting practices and value socialization. Countries exhibiting stronger cross-generational cultural influence consequently attract greater enrollment from such students.

This pattern is illustrated by the United States case analysis. American material products, commodities and visual arts culture have profoundly affected at least two generations of China. On the one hand, these young people who are studying abroad were born after 1980s and grew up completely after Reform and Opening, after the year of 1978, especially who were living in the cities such as Beijing and Shanghai. Their childhood lifestyles has been shaped by American products from all aspects, such as McDonald’s, Starbucks, Disney, Hollywood, Nike, P&G, NBA and so on (Li et al., 2024). Even the acquisition of American products has become a token reward mechanism for them if they make achievements in academics or being obedient to their elder family members (Xu, 2022). The American consumer culture which is a hyper-reality of being the ultimate luxury good for the other-directed and lifestyle full of efficiency, specialization and enjoyment through the body of daily supplies as well as entertainment commodities (Baudrillard, 1986, 1998) have already planted seeds in Chinese people and won their recognition deeply by generations.

Compared with American culture, British and European cultures, Japanese and Korean cultures, and Southeast Asian cultures have less significant influence on Chinese people’s daily consumption relatively. This makes the latter destined to be a niche, in China’s cultural landscape. The United Kingdom and Europe attracted the upper classes, who were obsessed with British aristocratic noble accents, gentleman etiquette and European luxury goods. Japanese and Korean culture is only popular among teenagers because of their comics and music. The culture of Southeast Asia is no more than natural scenery, food and affordable souvenirs, which is attracting the working class for tourism activities. However, American culture has influenced the lifestyles and aesthetic values on Chinese for generations in multifaceted, including consumption, work, study, socializing, and entertainment. SHR mentioned that the tendency for his parents to choose a study destination under cultural influence has been passed on to him.

“*My father’s first movie he watched was the Robocop. At that time, there were no movies of this type in Chinese market. He was shocked by the plot, dazzling movie stunts, and unbelievable budget of this movie. But he is older and full of work, so it is impossible to go to the Hollywood. It’s very proud that my dad let me to go to America experiencing the life he was longing for but cannot touch*.” (SHR-2407).

4.2.3 Other-directed Logic of Honor Producing: Life experience and social media.

While traditional frameworks emphasize the economic or academic returns of overseas education, this paper argues that an other-directed logic—wherein values and validation are sought from peers and reference groups (Riesman et al., 1950, p. 19)—now fundamentally structures this process. In this context, two interrelated forces have become central: the pursuit of a “high-quality life” as an experiential commodity, and the role of developed social media in enabling the real-time staging.

The concept of a “high-quality life” has evolved in consumer societies from the accumulation of material goods to the consumption of transformative experiences (Pine and Gilmore, 1999, pp. 56–59). For the other-directed individual, the value of an experience lies not only in experience itself but in its potential for narration, sharing, and social recognition. Studying abroad, therefore, becomes a privileged form of experiential consumption—a prolonged, immersive “life experience” that signals cosmopolitan taste, economic capital, and cultural fluency. This section posits that the level of development of a consumer and experience economy in a destination country directly influences its attractiveness as an honor-conferring choice.

The United States and the United Kingdom represent the apex of experience economy for students, offering not only elite education but also deeply branded lifestyle narratives. Europe offers high-quality living marked by rich cultural heritage, travel accessibility, and perceived work-life balance. Australia and New Zealand are branded around pristine environment and outdoor leisure. Asia offers proximity, academic excellence, and efficient urban living. Africa currently occupies a lower position in this experiential hierarchy in the minds of many middle-class families, as it is less consistently framed within a dominant global narrative of “high-quality living” as defined by contemporary consumption and safety paradigms, thus conferring less immediately recognizable social honor in an other-directed context. A respondent mentioned that the quality of life experience in the study destination is a key factor.

“*Yeah, the U.S. is still the top choice for me—it’s the whole culture, not just the degree. Think about the shopping scenes, the brand culture, the way you can experience everything from high-end boutiques to street-wear drops. Same goes for Europe. Living there means living inside that kind of aesthetic and consumption culture—it’s aspirational, and it looks good on a feed*.” (XCQ-2306).

Meanwhile, highly developed social media platforms provide infrastructure for the continuous, visually driven performance of life experience for honor producing (Pearce and Vitak, 2016). This section analyzes how Facebook, Instagram, and Twitter function as key arenas for honor manufacturing. Instagram is central for showcasing the aesthetic dimensions of the high-quality life. Facebook creates communities for validation and belonging. Twitter allows for the demonstration of cultural and intellectual immersion. The United States’ position as the most desirable destination is reinforced by a powerful synergy between its experiential offerings and its dominance of the social media landscape. American higher education is embedded in the very culture that produces the platforms upon which honor is performed.

This dynamic creates a seamless, self-reinforcing circuit. The United States provides a powerful, media-exported experiential package. Students then perform and narrate this experience primarily on U.S.-based, globally hegemonic social media platforms. The design and culture of these platforms naturally amplify the symbolic value of the American narrative. The resulting online visibility generates immediate peer validation, which concretizes social honor in real time. Consequently, choosing to study in the United States is synonymous with selecting the most highly visible and globally recognized stage for performing successful citizenship. SY believes that the level of social media development is one of factors in choosing a study destination, and she stated:

“*Around the world, the most popular social media platforms are mostly used by people in developed countries—you know, like Facebook, Twitter, and Instagram. That’s also where a lot of the well-known, influential users are based. So when someone like that likes, shares, or comments on your post, it’ s kind of a big deal—it feels like a real win.*” (SY-2306).

This part analyzes the stratified hierarchy of study-abroad destinations among China’s middle class, where the U.S. and U.K. dominate, followed by Canada/Western Europe, and then Australia/East Asia. This hierarchy functions as an honor producer, interpreted through the frameworks of tradition-directed, inner-directed, and other-directed social characters. The tradition-directed logic prioritizes pragmatic utility of foreign degrees within China’s collectivist context, where value is tied to enhancing family and community standing. The inner-directed logic highlights the intergenerational transmission of parental aspirations and cultural influence. Finally, the other-directed logic underscores the role of experiential consumption and social media performance, where destinations offering a recognizable “high-quality life” narrative.

### 4.3 Actors and shaping of honor production

Taking overseas education as an example, the production of social honor operates through distinct logics. For the middle class, it is not merely a form of conspicuous consumption or a type of cultural capital. Between these two dimensions, the middle class, as an agent in production of honor, flexibly selects how to interpret the meaning of study-abroad honor. This selective interpretation constitutes a typical socio-psychological habitus.

Through Riesman’s theory of social character, the logic of this habitus can be elaborated into three types: tradition-directed, inner-directed, and other-directed. However, these logics do not operate in isolation within specific social groups, rather, they appear in intertwined and nested forms across individuals and contexts. Using this perspective, we examine the various actors involved in the production of study-abroad honor, aiming to uncover the mechanisms through which such honor is constructed.

#### 4.3.1 Parents of international students: an inner-traditional-directed hybrid

Parents often function as an inner-traditional-directed hybrid. Their inner-directedness manifests in influencing destination choices based on personal aesthetic preferences and cultural affinity, reflecting a subjective valuation beyond mere utility. Concurrently, a strong traditional-directed orientation anchors their logic in pragmatism and collectivism, framing education as a strategic familial investment for tangible returns and inter-generational honor (Dada, 2025). This fusion results in a romanticized utilitarianism, where choices must satisfy both a personal vision of cultivated life and a traditional mandate for practical, family-advancing outcomes (Falk and Kosse, 2010).

This hybrid configuration, however, is not a static fusion but a site of ongoing negotiation and latent tension, particularly within the inter-generational dynamic (Qu and Fan, 2022). The parents’ inner-directed, culturally-romantic vision may conflict with the offspring’s more other-directed preoccupations with peer validation and experiential lifestyle consumption prevalent on social media. Consequently, the family becomes a micro-field where different logics of honor production are debated and reconciled. The resulting choice is often a negotiated compromise, strategically narrated to satisfy multiple audiences. MJY noted that his parents’ generation tends to choose study destinations based more on traditional values and personal intrinsic goals.

“*My mom chose the UK not only for its academic reputation but also because its cultural atmosphere matches our idea of what a good upbringing should be—that classic, refined vibe. Of course, we also did the math: a one-year master’s is time-efficient and cost-effective, and the degree is well-recognized by employers back home, especially in finance.”* (MJY-2408).

#### 4.3.2 The students themselves: an inner-other-directed hybrid

Students typically operate as an inner-other-directed hybrid. Their inner-direction is often circumscribed, accepting the overarching parental framework for studying abroad, yet asserts itself in a pronounced focus on subjective wellbeing, lifestyle quality, and personal experience during the sojourn. Simultaneously, a potent other-directedness drives a meticulous cultivation of digital identity, where the online cultural cachet and social media representativeness of the host country become paramount (Solmaz, 2020). Their behavior is a negotiated performance, balancing compliance with familial strategy and the pursuit of peer-validated, socially visible capital.

This hybridity requires constant negotiation across social fields, leading to strategic self-presentation. Students emphasize self-growth to family, while performing a curated, Instagram-ready lifestyle to peers, showcasing cosmopolitan consumption. Algorithmic platforms mediate this performance, actively shaping visibility standards and intensifying pressure to conform to trends. The study abroad experience thus becomes a liquid form of experiential capital (Zhang, 2023). Its conversion into lasting social honor, however, hinges on validation from the very audiences that shaped it: familial approval and peer recognition. Therefore, student agency involves navigating, not resolving, these tensions to profit from them. A student studying in the UK mentioned that her decision to study abroad followed their parents’ guidance, while also considering the quality of life there.

*“Honestly, my family chose my major—business, for practicality. But the city was my call: London. There are so many museums, concerts, and the lifestyle here is totally different. Plus, my Instagram and TikTok are full of great content—my friends say my feed looks like a travel blogger’s.”* (GJP-2306).

#### 4.3.3 Governments and corporations: an other-traditional-directed hybrid

Institutional actors like governments and corporations exhibit an other-traditional-directed hybrid logic. Their other-directedness is evident in converting recruitment metrics of elite graduates into departmental performance and collective honor, directly responding to competitive rankings and market expectations. This intertwines with a deep-seated traditional-directed reverence for institutional pedigree, reducing complex meritocratic assessment to a symbolic fetishism of elite diplomas (Collins, 1979, pp. 35–40). This synergy creates a powerful, reductive market signal that rigidly equates prestige with competence.

This hybrid institutional logic acts as a core engine of social reproduction, solidifying elite universities as the chief arbiters of symbolic capital (Bourdieu, 1986, pp. 241–258). It creates a self-reinforcing cycle where institutional prestige validates graduates, whose employment, in turn, reaffirms that prestige. In the digital era, this cycle is amplified as platforms like LinkedIn algorithmically quantify and automate the filtering based on prestige, masking a social judgment with technical neutrality. However, the system harbors tensions: the demand for innovative talent can clash with a reverence for pedigree, potentially creating niches for non-traditional skills. Moreover, equating prestige with competence risks credential inflation, workforce homogenization, and the neglect of capable non-elite graduates, exposing a contradiction between meritocratic ideals and practices of exclusion. Thus, while stabilizing status hierarchies in the short term, this logic plants the seeds for its own recurring crises of legitimacy and efficiency. One respondent noted that governments and enterprises tend to place greater emphasis on the collective honor and public comments associated with overseas diplomas.

“*Top institutions prioritize graduates from top-50 global universities right from the resume screening stage. They do not take it as discrimination but efficiency. They think hiring someone from Ivy League school is a win for their department’s performance*” (XCQ-2305).

#### 4.3.4 Relatives and peers: an other-traditional-directed social audience

The extended social network of relatives and peers acts as an other-traditional-directed audience. Their other-directedness fuels conspicuous comparison and status gossip, making educational pathways a key currency in social face competition (Stranges et al., 2019). This is layered with a traditional-directed, utilitarian calculus that constantly evaluates the “cost-effectiveness” and return on investment of the endeavor. They thus apply a dual-pressure system, demanding choices that are simultaneously prestigious for display and defensible on practical grounds.

The dual-pressure system exerted by this external audience actively structures the affective and moral landscape of educational migration. It imposes a distinct performative burden on students and families, compelling them to narrate their choices strategically—emphasizing experiential prestige for peers and practical utility for elders. This pressure becomes internalized, shaping the actors’ habitus (Reay et al., 2001). Fears of gossip and investment failure then powerfully constrain “legitimate” choices to a narrow range of high-status, defensible destinations. Thus, the audience functions as a key mechanism of social control, translating cultural logics into daily pressures that reproduce value hierarchies and channel aspirations into sanctioned pathways. A participant mentioned that their relatives care more about how the choice of study destination impacts collective honor and the opinions of others.

“*My uncle think UCB is really impressive even with a cost of around 700,000 RMB a year. This is a huge success to show off in my whole family. Having that kind of background even helps in the dating market.*” (XWS-2507).

#### 4.3.5 Honor as a negotiated consensus

Consequently, the production of study-abroad honor is not governed by a monolithic logic. Instead, it arises from the strategic interactions and negotiations among hybrid social actors. This process generates a dynamic and contested consensus, forged at the intersection of internalized values, traditional imperatives, and other-directed social markets (Qi et al., 2022).

This complex interplay of competing logics elucidates the characteristic anxiety and calculative rationality pervading contemporary educational migration (Wu and Hou, 2024). Individuals and families must navigate a dense web of competing significations—emotional, utilitarian, social, and experiential. Their objective is to secure a provisional yet socially legitimized position within this symbolic economy of honor. Thus, honor is not merely acquired but is continuously performed and validated through a strategic alignment of personal agency with overlapping structural and normative forces (Üstüner and Thompson, 2012). This analytical framework suggests that studying abroad is not merely an educational choice, but rather a practice of honor production that involves multiple actors and competing logics. It profoundly reflects the complex interplay between the individual and the collective, tradition and modernity, within a context of social transformation.

This part conceptualizes the production of study-abroad honor as a negotiated consensus emerging from strategic interactions of hybrid social actors. Parents often embody an inner-traditional-directed logic, merging personal cultural affinity with utilitarian family strategy. Students typically navigate an inner-other-directed hybrid, balancing parental expectations with a curated, peer-oriented performance of experience. Institutions and the broader social audience operate with an other-traditional-directed logic, fetishizing elite pedigree for competitive signaling while demanding practical returns. The resulting social honor is not predetermined but is dynamically forged at intersection of these competing logics—traditional, inner-directed, and other-directed—highlighting educational migration as a contested field where personal agency meets structural forces in continual manufacture of honor.

## 5 Conclusions and discussions

This study has sought to unravel complex socio-psychological mechanisms underpinning the production of social honor within China’ s middle class, via a selection of overseas study destinations as an empirical lens. Moving beyond frameworks of economic calculation, we have argued that this phenomenon constitutes a strategic field where honor is actively manufactured through the interplay of multiple logics and actors. Drawing on Riesman’s theory of social character, our analysis reveals that Chinese middle class navigates this field not through a singular logic but through a hybrid social-psychological habitus from different social characters. This hybridity encompasses tradition-directed imperatives, such as familial duty and pragmatic return. It also includes inner-directed pursuits of self-cultivation, alongside other-directed performances that are calibrated for social media visibility and peer recognition.

Key actors, including students, parents, institutions, and social networks, each operate from distinct yet overlapping positional logics. In this process, their continuous negotiation collectively forges a dynamic and often contested consensus on valuable social honor. This process operates within China’s “Compressed Modernity” (Kyung-Sup, 2022, pp. 19–21), which forces the juxtaposition of agricultural, industrial, and informational-era values. The strategic navigation of these competing logics thus defines contemporary middle-class identity work.

Our findings engage in critical dialogue with two dominant theoretical traditions in the study of status and distinction: Veblen’ s conspicuous consumption and Bourdieu’s cultural capital. In contrast to Veblen’ s model, where consumption primarily signals status to a generalized audience, the honor production we observe is more nuanced and multidirectional. Conspicuous expenditure on overseas education is undeniably present, but its meaning is not stable. It can be downplayed as a pragmatic investment, framed as an indispensable experience for self-realization, or showcased as part of a digital lifestyle (Besta et al., 2025). The conspicuousness itself is strategically modulated depending on the audience and the aspect of honor being emphasized. Thus, consumption is not an end in itself but a flexible resource deployed within a broader repertoire of honor-making strategies (Currid-Halkett, 2017, pp. 29–33).

Similarly, our framework extends and complicates Bourdieu’s concept of cultural capital. While a degree from a prestigious foreign university functions as institutionalized cultural capital, our analysis illuminates the process of its acquisition as a field of struggle and performance. Bourdieu’s model emphasizes the tacit, embodied nature of cultural capital acquired through familial socialization. However, in the context of transnational education, we see an accelerated, intentional, and highly mediated process of acquisition (Choi and Lim, 2024). The cultural capital is not merely inherited; it is painstakingly project-managed by families.

The hybrid character model explains why cultural capital, like a foreign degree, can be legitimized through different stories. Firstly, it can be narrated as a vehicle for family uplift, converting it into social capital. Secondly, it can be framed as a testament to personal grit, thereby reinforcing an inner-directed identity. Thirdly, it can be presented as a badge of trendy global citizenship, enhancing symbolic capital in digital networks. Therefore, we argue that the focus should shift from cultural capital as a possessed asset to cultural capital-in-the-making, a process governed by the strategic logic of hybrid social character.

The primary theoretical innovation of this paper lies in synthesizing Riesman et al.’s (1950, p. 39) social character typology with contemporary studies of globalization and digital society to propose hybrid social characters as a novel analytical framework. This framework transcends limitations of applying singular Western-centric models to a rapidly transforming society like China. It captures simultaneous, often contradictory, pulls of filial piety, self-development, and social media validation that define the modern Chinese middle-class experience.

Secondly, we contribute to the sociology of education and migration by reconceptualizing international student mobility as a performative project of honor production (Spangler, 2025). Specifically, we show how destinations are hierarchically coded and how choices are embedded in intergenerational strategies and digital self-presentation. At the micro-level, we provide a mechanism for this process: the negotiated interactions among actors wielding different logics explain how macro-structural forces like compressed modernity are enacted in everyday life.

At Last, while this study is grounded in the specific context of the Chinese middle class, the analytical framework of hybrid social character developed here offers a portable lens for examining status production and educational strategies in other rapidly transforming societies. The findings are not merely descriptive of a Chinese phenomenon but illuminate a broader social process: how groups navigate status anxiety and identity projects within contexts of compressed modernity (Oleksiyenko et al., 2024). Middle and aspirational classes in other emerging economies (e.g., India, South Korea, Brazil, Gulf states) also engage in global educational markets, creating their own stratified hierarchies of desirable destinations. The theoretical value of this case lies in providing a model to decode howthese hierarchies are made meaningful—not just as economic calculations, but as sites where competing cultural logics of worth are played out (Akkaş and Aksakal, 2025). Future comparative research could test how the weight and interplay of the three logics differ in societies with varying strengths of individualism, collectivism, or colonial histories.

This study has several limitations that point to avenues for future research. First, our sample primarily captures the perspectives of students and parents with means to consider overseas education. While insightful, it potentially overlooks those for whom this path is inconceivable or less successful participants in this honor practice. Second, the research design is qualitative and focused on China. Comparative studies with other rapidly developing societies could test generalizability of the hybrid character model. Lastly, the study offers a snapshot in a rapidly evolving field. Longitudinal research is crucial to trace how these honor-production strategies evolve with shifting global politics, economic climates, and domestic education policies.

## Data Availability

The original contributions presented in the study are included in the article, further inquiries can be directed to the corresponding author at hankdu123@163.com.

## Footnotes

1
https://www.iie.org/Research-and-Insights/Open-Doors/Data/International-Students/Places-of-Origin

2 IIE Open Doors: https://opendoorsdata.org/

3 It originates from the poem *Ode to The Prodigy* (《神童诗》), written by Wang Zhu of the Song Dynasty (960–1279 CE) in China.

4

$\begin{array}{l} \text{Extract coding convention}:\text{the first three letters represent}\;{\text{interviewee}}^{’}s\;\text{name},\\ \text{and the following four digits indicate the year and month that interview was}\;\\ \text{conducted}. \end{array}$
